# Microsatellite diversity and complexity in the viral genomes of the family Caliciviridae

**DOI:** 10.1186/s43141-023-00582-x

**Published:** 2023-11-24

**Authors:** Md Gulam Jilani, Mehboob Hoque, Safdar Ali

**Affiliations:** 1 Department of Biological Sciences, Clinical and Applied Genomics (CAG) Laboratory, Aliah University, IIA/27, Newtown, Kolkata, 700160 India; 2 Department of Biological Sciences, Applied Bio-Chemistry (ABC) Lab, Aliah University, Kolkata, India

**Keywords:** Caliciviridae, Simple sequence repeats, MISA, Incidence, Prevalence, Phylogenetics

## Abstract

**Background:**

Microsatellites or simple sequence repeats (SSR) consist of 1–6 nucleotide motifs of DNA or RNA which are ubiquitously present in tandem repeated sequences across genome in viruses: prokaryotes and eukaryotes. They may be localized to both the coding and non-coding regions. SSRs play an important role in replication, gene regulation, transcription, and protein function. The Caliciviridae (CLV) family of viruses have ss-RNA, non-enveloped, icosahedral symmetry 27–35 nm in diameter in size. The size of the genome lies between 6.4 and 8.6 kb.

**Results:**

The incidence, composition, diversity, complexity, and host range of different microsatellites in 62 representatives of the family of Caliciviridae were systematically analyzed. The full-length genome sequences were assessed from NCBI (https://www.ncbi.nlm.nih.gov), and microsatellites were extracted through MISA software. The average genome size is about 7538 bp ranging from 6273 (CLV61) to 8798 (CLV47) bp. The average GC content of the genomes was ~ 51%. There are a total of 1317 SSRs and 53 cSSRs in the studied genomes. CLV 41 and CLV 49 contain the highest and lowest value of SSRs with 32 and 10 respectively, while CLV16 had maximum cSSR incidence of 4. There were 29 species which do not contain any cSSR. The incidence of mono-, di-, and tri-nucleotide SSRs was 219, 884, and 206, respectively. The most prevalent mono-, di-, and tri-nucleotide repeat motifs were “C” (126 SSRs), AC/CA (240 SSRs), and TGA/ACT (23 SSRs), respectively. Most of the SSRs and cSSRs are biased toward the coding region with a minimum of ~ 90% incident SSRs in the genomes’ coding region. Viruses with similar host are found close to each other on the phylogenetic tree suggesting virus host being one of the driving forces for their evolution.

**Conclusions:**

The Caliciviridae genomes does not conform to any pattern of SSR signature in terms of incidence, composition, and localization. This unique property of SSR plays an important role in viral evolution. Clustering of similar host in the phylogenetic tree is the evidence of the uniqueness of SSR signature.

**Supplementary Information:**

The online version contains supplementary material available at 10.1186/s43141-023-00582-x.

## Background

The Caliciviridae family are non-enveloped viruses, about 27–35 nm in diameter with an icosahedral symmetry and ss-RNA (6.4–8.6 kb) as a genetic material. Their genomes contain multiple ORFs/genes encoding for structure and non-structure protein. The Caliciviridae family has six established genera (*Norovirus, Sapovirus, Lagovirus, Vesivirus, Nebovirus,* and *Recovirus*) and five more genera have been proposed (*Valovirus, Nacovirus, Bavovirus, Minovirus*, and *Salovirus*). The virus members of the family Caliciviridae are known to have a wide range of hosts including human, geese, yellowfin seabream, greater green snake, arctic lamprey, frogs, and birds. These viruses are associated with several diseases like digestive tract infections, vesicular lesions, reproductive failure, stomatitis, upper respiratory tract and systemic diseases, and hemorrhagic disease [1–7]. Some members of the genus *Norovirus* and *Sapovirus* are the major causative agent for human epidemic acute gastroenteritis around the world. Their transmission happens through both direct contact as well as indirect means like fecal matter, vomitus or respiratory secretions, contaminated food, water, and fomites [8].

A complete understanding of the Caliciviridae genomics will be beneficial for the management of the problems related to not only these viruses but others as well. One of the tools that has been used extensively for exploring of genomes has been the microsatellites or simple sequence repeat (SSR). These are short tandem repeats of 1–6 bp repeat motif reportedly exhibiting ubiquitous presence in both the coding as well as non-coding regions of the prokaryotic and eukaryotic genomes [9, 10]. SSRs are known to play an important role in replication, gene regulation, transcription, and protein function. They are also sites for generating genome diversity and hence not only contributing but driving of viral genome evolution. The present study focusses on the distribution, incidence, composition, and microsatellites across Caliciviridae genomes.

## Methods

### Retrieval of genome sequences

The complete genome sequences of 62 Calicivirus (CLV) genomes of the family Caliciviridae which is listed in ICTV (https://ictv.global/report_9th/RNApos/Caliciviridae) were retrieved from the NCBI database (http://www.ncbi.nlm.nih.gov/) and saved in GenBank information data and FASTA formats. The Virus-Host Database (https://www.genome.jp/virushostdb/note.html) was used for elucidating virus-host information. The summary of sequences analyzed in the study has been provided in Supplementary file 1.

### Extraction of microsatellites and analysis

The extraction of microsatellites was done using MISA software (https://webblast.ipk-gatersleben.de/misa/) with previously standardized parameters for viral genomes [10, 11]. Briefly, mono- to hexa-nucleotide repeat motifs were extracted with permissible minimum repeat size as follows: 6 (mono-), 3 (di-), 3 (tri-), 3 (tetra-), 3 (penta-), 3 (hexa-). Compound microsatellites (cSSR) were also identified. cSSR is the incidence of multiple SSRs separated by a maximum allowed distance (dMAX). The cSSRs were extracted with varying dMAX (10, 20, 30, 40, 50) to study the clustering of SSRs in the studied genomes. All other parameters were used as default. The data of microsatellites obtained was exported and saved in Microsoft Excel 2016 for further analysis which included relative abundance (RA) and relative density (RD); cSSR%; SSR prevalence; motif composition; tract size; and localization across coding and non-coding regions of the studied genomes.

### Phylogenetic analysis

ETE3 v3.1.1 GenomeNet (https://www.genome.jp/tools-bin/ete) tool was used for alignment and phylogenetic tree building as per standard protocols [12]. The sequences were aligned using MAFFT v6.861b with the set parameters [13]. Gappyout algorithm of trimAl v1.4.rev6 was performed for pruning of alignment [14]. To analyze the alignment perfectly match in terms of evolutionary, ML tree inference among JC, K80, TrNef, TPM1, TPM2, TPM3, TIM1ef, TIM2ef, TIM3ef, TVMef, SYM, F81, HKY, TrN, TPM1uf, TPM2uf, TPM3uf, TIM1, TIM2, TIM3, TVM, and GTR models using pmodeltest v1.4. ML tree was inferred using RAxML v8.1.20 ran with model GTRGAMMA and default parameters [15]. ML tree were maintained with the 100 bootstrapped trees. Interactive Tree Of Life (ITOL) webtool is used for the phylogenetic tree annotation and visualization [16].

### Correlation analysis

Correlation analysis was performed to determine the impact of genome features like size and GC content on various parameters associated with microsatellites like incidence, RA, RD, and cSSR%. Simple linear regression analysis was performed using Microsoft Office Excel 2016 for the same.

### Software and tools for illustrations

All the figures are generated by Flourish (https://flourish.studio/) and RAWGraphs 2.0 (https://app.rawgraphs.io/), Gene structure display server (http://gsds.gao-lab.org/), and Microsoft PowerPoint 2016. All the tools are online webserver, and the figures are generated by default parameters. Gene structure display server required FASTA, GTF/GFF3, or BED format file for input data.

**Fig. 1:**
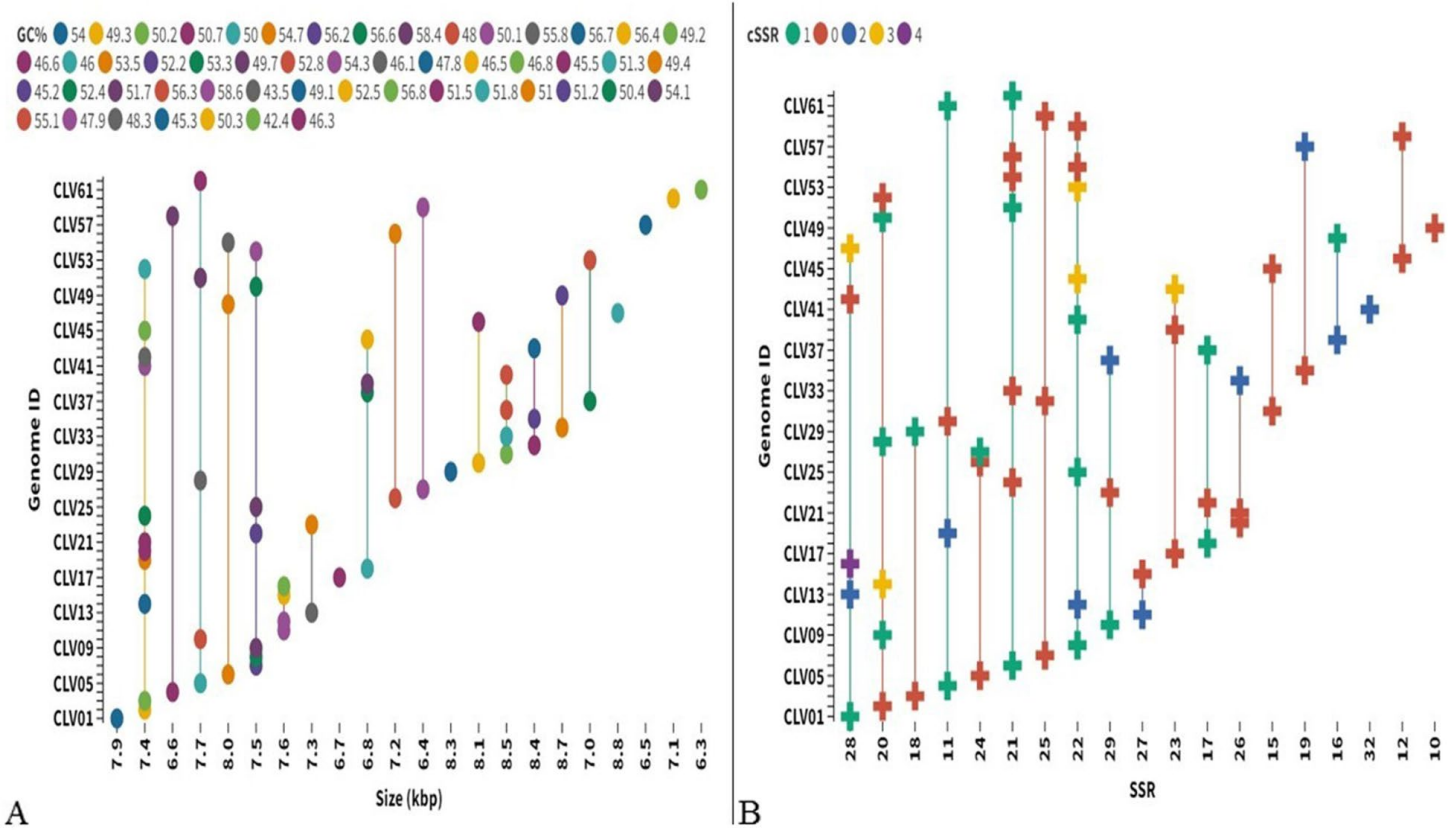
Genome features and microsatellite incidence. **A** Size of genome and GC content. *X* axis represents the genome size, *Y* axis has genome ID and the circle designates the GC%. Each circle is a specific genome and all circles in the same line have the same genome size with varying GC% designated by their positioning on the line. **B** Incidence of SSR and cSSR. Note the diversity in genome features as well as microsatellite incidence. The *X* axis represents SSR incidence while there were 0 to 4 cSSRs per genome indicated by different colors. Multiple plus signs on the same straight line suggest varying cSSR incidence with same number of SSRs

**Fig. 2:**
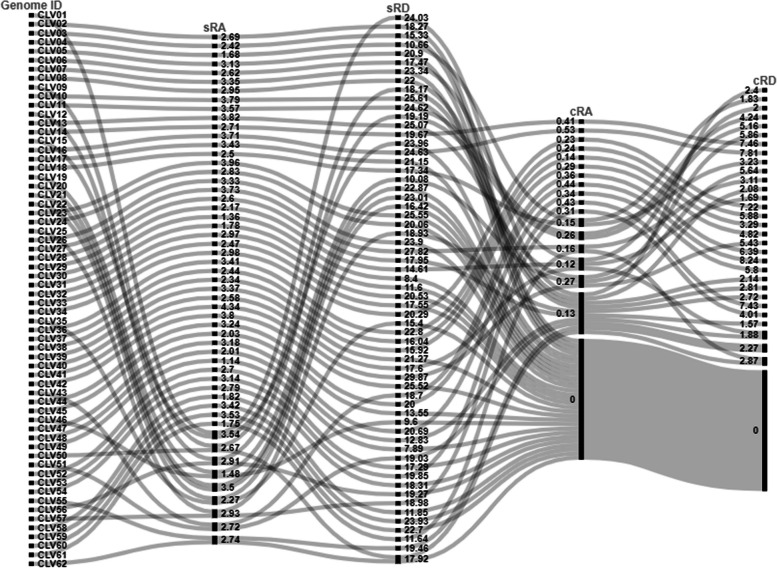
Relative abundance (RA) and relative density (RD) of incident SSRs and cSSRs. sRA and sRD represent RA and RD for SSR. cRA and cRD represent RA and RD for cSSR. The lines interlink genome ids with the various RA and Rd values

**Fig. 3:**
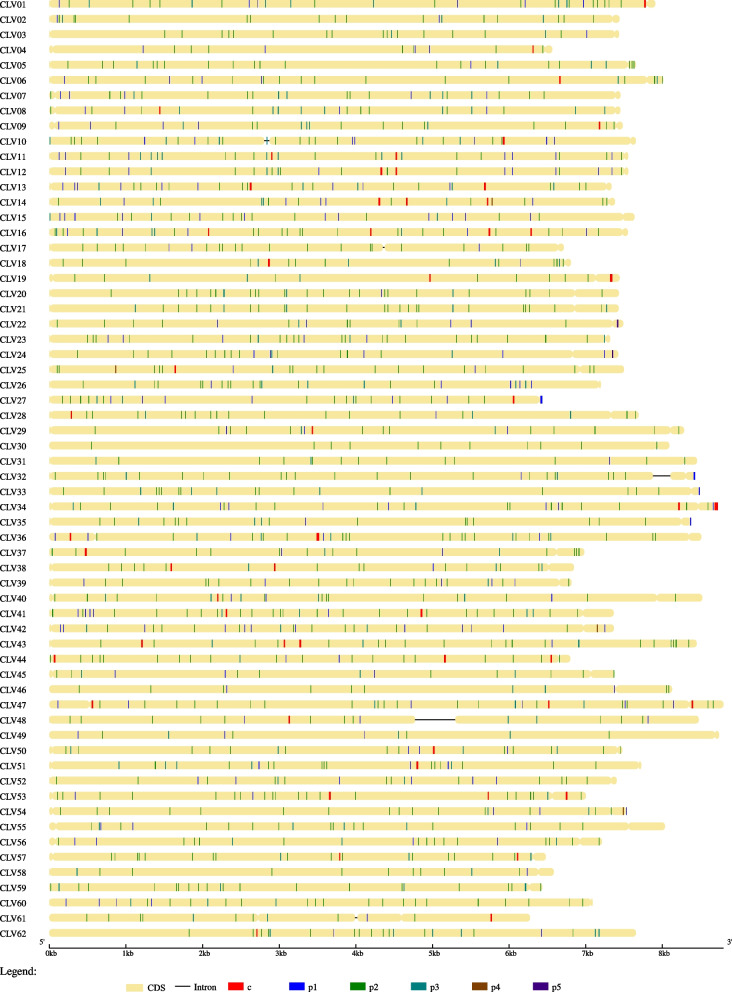
Overview of microsatellite map of the *Caliciviridae* genomes. Note the uniqueness of microsatellite signature of each genome. The coding and non-coding regions are represented by bars and straight line respectively. The mono- to penta-nucleotide SSRs and cSSRs are shown by different color intersections on the bar. A color key has been provided at the bottom for reference

**Fig. 4:**
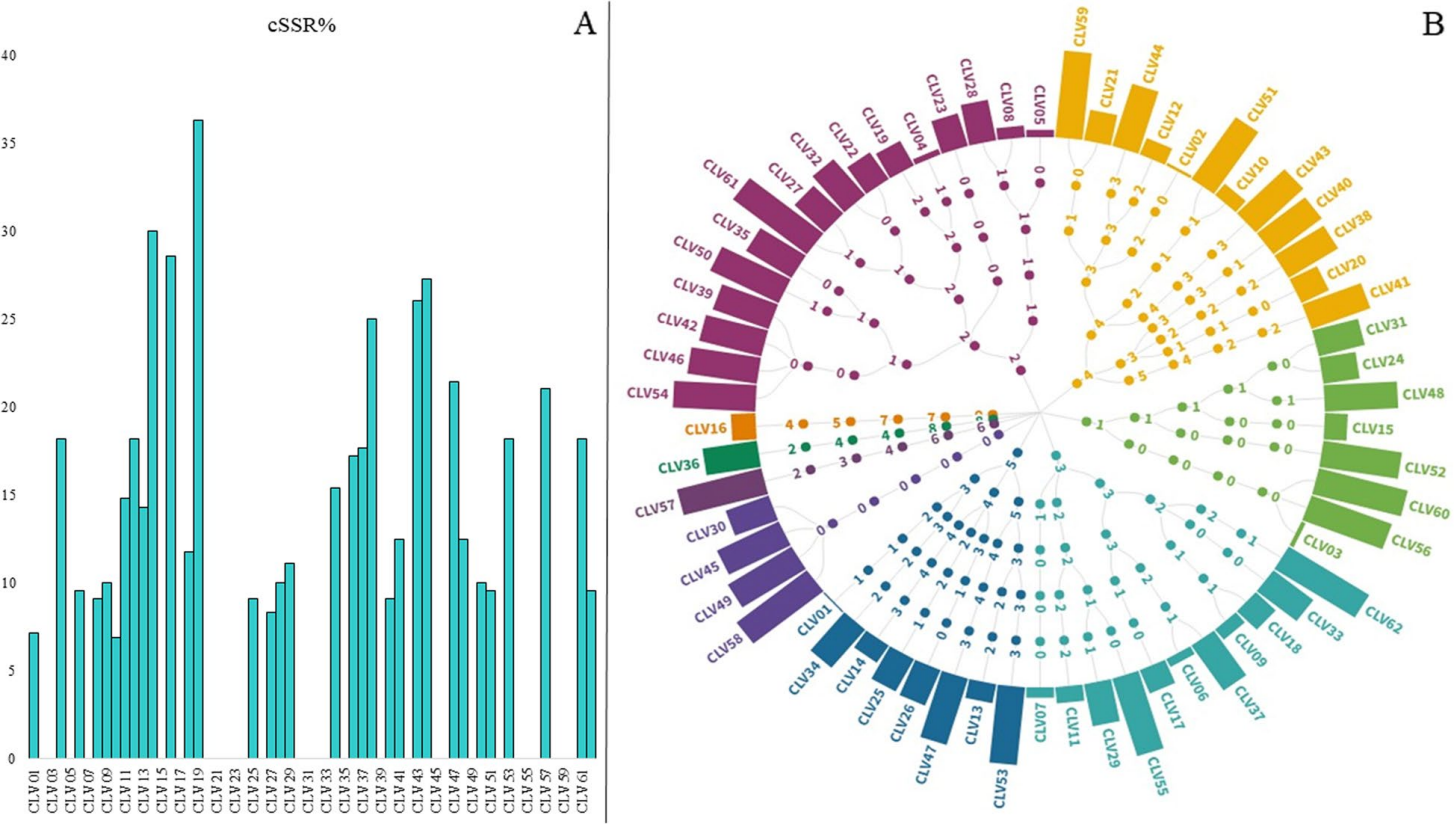
cSSR analysis in the Caliciviridae genomes. **A** The percentage of SSRs present as a part of cSSR (cSSR%). **B** Variation in incidence of cSSRs with increasing dMAX. Each dot represents an incidence at a particular dMAX. The line from genome ID to the center represents the path of increasing dMAX and corresponding cSSR incidence, details of which are provided in Supplementary file 1

**Fig. 5:**
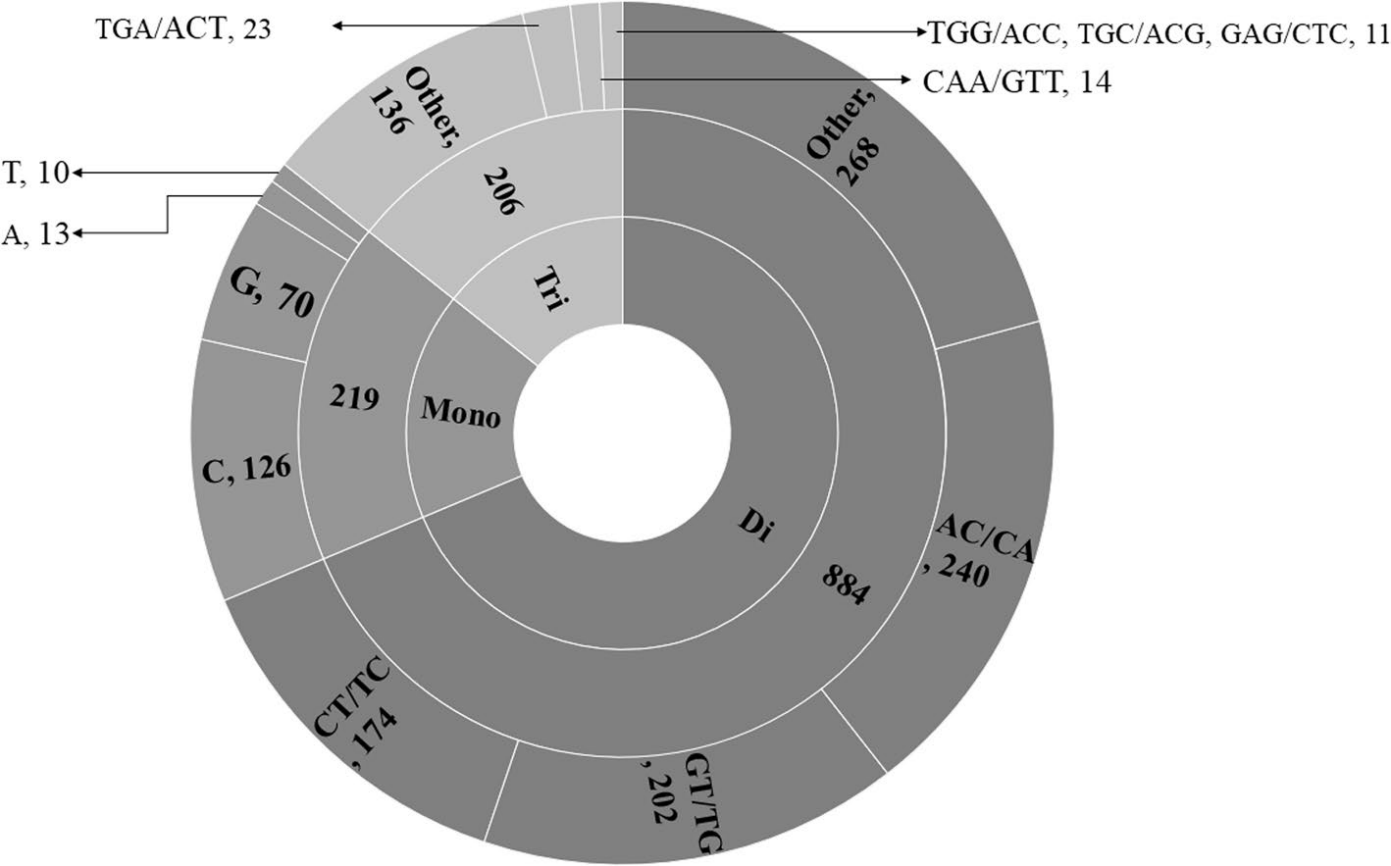
Motif composition of mono-, di- and tri-nucleotide Caliciviridae genomes. The most prevalent motifs in each of mono- to tri-nucleotide repeats are shown

**Fig. 6:**
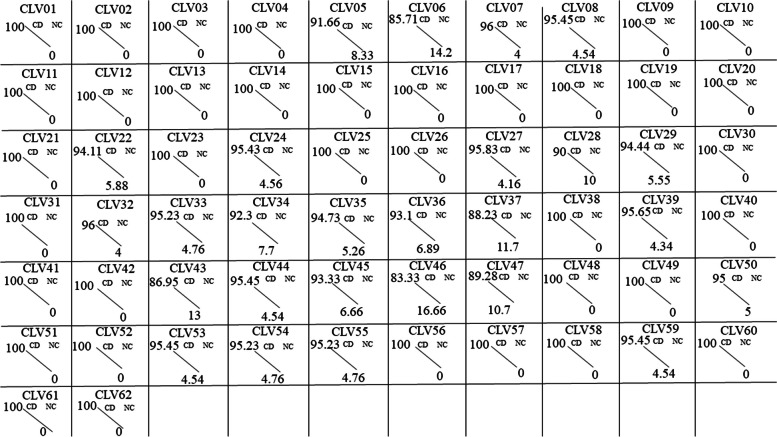
Localization of microsatellites across the genomes’ coding and non-coding regions. The percentage of SSRs present in coding region (CD) and non-coding (NC) are represented at the two ends of line for each genome. The genomes having all SSRs present in the coding region are depicted by blue line, whereas other colored lines represent differential distribution of SSRs across coding and non-coding regions

**Fig. 7:**
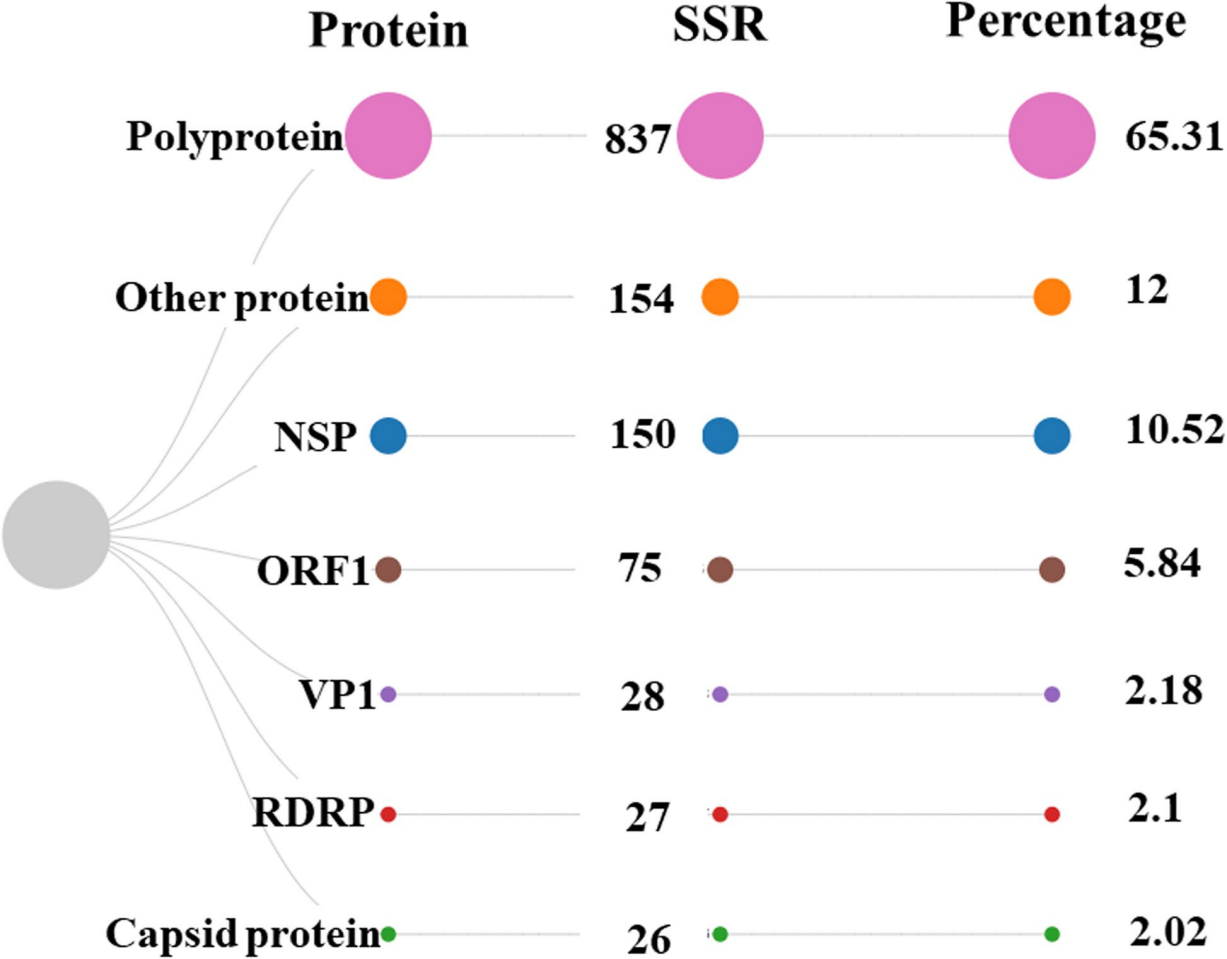
Distribution of SSRs across the different proteins. Only the proteins with most SSRs have been represented for the sake of clarity, while the rest have been shown as others

Figure 1 is Dot connector (the same genome size and SSR incidence are connected with line); Fig. 2 is Alluvial Diagram (genomes with the same sRA, sRD, cRA, and cRD are grouped and connected by line); Fig. 3 is generated by Gene structure display server; Fig. 4A and B is Column chart and Radial tree (genomes with the same dMAX50 are placed in the same group in radial tree); Fig. 5A and B is Sunburst chart and Column chart; Fig. 6 is flow chart (percentage of SSR in coding and non-coding in the studied genomes); and Fig. 7 is Linear dendrogram. All the figures are generated on May 5, 2022.

## Results

### Genome features

The mean genome size of Caliciviridae genomes was about 7538 bp with a minimum and maximum value of 6273 (CLV61) to 8798 (CLV47) bp. The average GC content of the genomes was ~ 51% with a range from 42.4% (CLV61) to 58.6% (CLV41). The genome size and GC% of the Calicivirus genomes have been shown in Fig. 1A while the details have been provided in Supplementary file 1. While a total of 11 genomes had a size of 7.4 kb, the GC% was much more variable.

### Microsatellite incidence

The extraction revealed a total of 1315 SSRs and 55 cSSRs from the Caliciviridae genomes. The SSR incidence ranged from 32 (CLV41) to 10 (CLV49) with an average of 21 SSRs per genome. Interestingly, there appears no linearity between genome size and SSR incidence as exemplified by CLV41 with genome length of 7365 bp having 32 SSRs, while CLV49 with 8741 bp genome had just 10 SSRs. The incidence of SSR and cSSR has been represented in Fig. 1B, summarized in Supplementary file 1, and details in Supplementary file 2.

We assessed the possible influence of genome size and GC content on number of SSR and cSSR incidence, RA, RD, of SSRs and cSSRs and cSSR% in SSR. Genome size of the assessed CLV type has no positive significance on any number. All are showing non-significant correlation of SSR (*r* = 0.014075; *p* = 0.358409), cSSR (*r* = 0.000491; *p* = 0.864181), sRA (*r* = 0.035470; *p* = 0.142668), sRD (*r* = 0.028883; *p* = 0.186633), cRA (*r* = 0.003523; *p* = 0.646749), cRD (*r* = 8.95E- 06; *p* = 0.981590), and cSSR% (*r* = 0.009579; *p* = 0.449164) while GC content also did not show positive and significance on any number, all the numbers show negative and non-significant correlation of SSR (*r* = 0.035370; *p* = 0.143240), cSSR (*r* = 0.028945; *p* = 0.186158), sRA (*r* = 0.036195; *p* = 0.138573), sRD (*r* = 0.049552; *p* = 0.082033), cRA (*r* = *0.027536; *p* = 0.197401), cRD (*r* = 0.038966; *p* = 0.124079), and cSSR% (*r* = 0.017160; *p* = 0.310163).

We further looked at the distribution of microsatellites and their iteration length. Relative abundance (RA) is defined as the number of microsatellites present per kb of the genome, whereas relative density (RD) is the number of bases present as SSR per kb of the genome. A higher RA value would denote greater SSR incident frequency, whereas a higher RD value would indicate a greater number of iterations and hence higher tract size of SSRs. The RA and RD for SSR in the Caliciviridae genomes ranged from 1.14 and 7.89 (CLV49) to 4.34 and 29.87 (CLV41) respectively. The cSSR incidence had a maximum of 4 in CLV16. A total of 29 species had no cSSR in their genomes (Fig. 2, Supplementary file 1). These genomes with no cSSR incidence had an SSR incidence range from 10 to 29 (Fig. 1B). The incidence of SSRs and cSSRs have been summarized in Fig. 3.

Owing to the variant cSSR incidence, we further dwelled into it through two features: First, the presence of SSRs present as a part of cSSR represented as a percentage of total SSRs, also known as cSSR%; secondly, the impact of increasing dMAX on the incidence of cSSRs. The cSSR% for all the 29 genomes with no cSSR was zero. For the other species, it ranged from 6.89 (CLV10) to 36.36 (CLV19). This implies that in CLV19, more than one-third of the incident SSRs have another SSR in their vicinity (Fig. 4A, Supplementary file 1). What should also be mentioned here is the fact that CLV10 is among the genomes with a lesser incidence of 11 SSRs. Contrastingly, CLV41 with the highest incidence of 32 SSRs had a cSSR% of 12.5. This data was pertaining to cSSRs with dMAX of 10. In order to assess if more SSRs are present in adjoining regions of observed cSSRs, we increased dMAX at intervals of 10 up to a maximum of 50. Expectedly, the increase in the dMAX value led to a greater number of cSSRs but the increase in cSSR incidence does not follow any priority principle as shown in Fig. 4B and Supplementary file 1.

### Microsatellite composition

The composition of microsatellites can be studied through three aspects namely motif length (mono- to hexa-); motif constitution (A/T/G/C) and tract size (number of repeats). In terms of motif length, the SSRs were predominantly composed of mono- to tri-nucleotide SSRs. There were only four (CLV14, CLV25, CLV42, CLV54) and two (CLV22, CLV 24) tetra- and penta-nucleotide repeats observed across the genomes. No hexa-nucleotide repeats were observed (Supplementary file 2). Since the tetra- to hexa-nucleotide repeats were rarely incident for further discussion about motif composition, only mono- to tri-nucleotide repeats were considered.

The motif composition of the SSR of the studied genomes has been represented in Fig. 5. All the detail has been mentioned in Supplementary files 2 and 3. As the studied genomes were rich in GC content the same is somewhat reflected in repeat motifs. Among the mono-nucleotide repeats, “C” is the most prevalent motif comprising of around 58% (126 of 219) followed by “G” (70 incidences). The other two motifs herein were almost equally represented with “A” (13) and “T” (10) incidences. Similarly, the most prevalent motif composition of di-nucleotide repeats was AC/CA comprising around 27% (240 of 884) followed by GT/TG (202 of 884), while TGA/ACT is the most prevalent motif of tri-nucleotide repeat incidence.

The tract size of mono- to tri-nucleotide repeats has been revealed that most of the genomes have the highest tract size contributed by di-nucleotide repeats (Supplementary file 3). Fifty-eight species have di-nucleotide tract size as the maximum followed by two with mono- and tri-nucleotide tract size. CLV41 and CLV20 have the highest tract size of 148 and 134 bases from di-nucleotide repeats respectively. Interestingly, there are eight species with the same number of SSR incidence. CLV8, CLV12, CLV25, CLV40, CLV44, CLV53, CLV55, and CLV59 have the same number of 22 SSRs present in their genomes (Fig. 1, Supplementary file 1), but their tract size is highly variant. CLV12, CLV25, CLV40, CV44, CV53, CV55, and CLV59 have the maximum tract size from di-nucleotide repeats with 68 bases (11 SSRs), 108 bases (18 SSRs), 86 bases (14 SSRs), 114 bases (19 SSRs), 118 bases (11 SSRs), 90 bases (15 SSRs), and 112 bases (18 SSRs) respectively, whereas CLV8 has the highest tract from tri-nucleotide repeat with 84 bases (9 SSRs).

### Microsatellite distribution

The distribution of SSRs across the genome was analyzed at two different levels. First, overall distribution between coding and non-coding regions. This has to be analyzed with caution as the viral genomes are predominantly coding. A total of 36 genomes had no non-coding region (Supplementary file 4). The genome-wise distribution of microsatellites in coding regions has been shown in Fig. 6, and details are provided in Supplementary files 2 and 4. Evidently, the 36 genomes lack any non-coding SSRs as the genome is fully coding.

Thereon, we analyzed the protein-specific localization of the SSR distribution. It revealed that 65% of SSR (837) is localized in the polyprotein region followed by non-structural protein in the second position containing 10% of SSR (150) as represented in Fig. 7. We also studied the SSR density of various proteins across genomes. The protein/ORF with the maximum and minimum microsatellite density has been mentioned in Supplementary file 4.

### Phylogenetic analysis

**Fig. 8:**
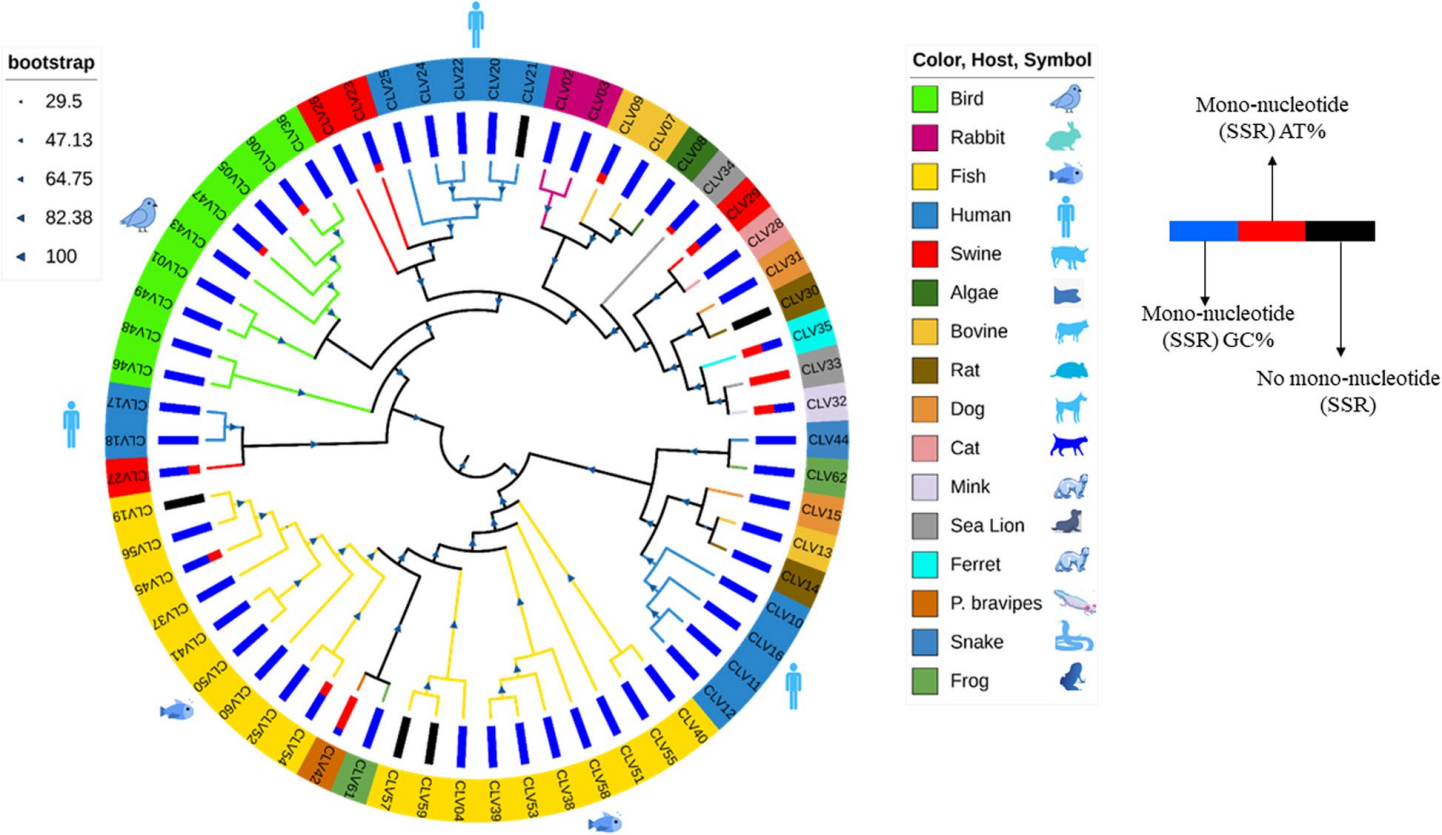
Phylogenetic analysis of the Caliciviridae genomes. The host and localization of mono-nucleotide repeats in the A/T region of the genome have also been depicted in a color-coded manner with key provided

In order to understand the evolutionary relationship between the members of Caliciviridae, the phylogenetic tree was constructed and annotated with some specific aspects, which has been represented in Fig. 8. Phylogenetic tree has been analyzed in two different ways. Firstly, the host range of the viruses. As clear in Fig. 8, the viruses which have the same or similar host are in close proximity on the tree demonstrating the virus host being one of the driving forces for evolution. Secondly, we analyze the phylogenetic tree between the host of the virus and mono-nucleotide SSR repeats.

## Discussion

Genome-wide scan study revealed genome size, GC content, occurrence, abundance, and composition of SSRs and cSSRs tracts across 62 Caliciviridae genomes. The average length of genome of Caliciviridae was about 7538 bp with a maximum and minimum of 6273 to 8798 bp. The average GC content of the genomes was ~ 51% with a range from 42.4 to 58.6%. This is one of the richer set of genomes in terms of GC% and would be interesting to observe the implications on microsatellite particularly in terms of motif composition. A total of 1315 SSRs and 55 cSSRs have been retrieved from the Caliciviridae genome. The average SSR per genome is 21 with a range from 32 to 10. The incidence of cSSR ranged from 0 to 4. The SSR incidence is independent from genome size. For instance, CLV13 has genome size (7338 bp) with 28 SSR incidence, while CLV31 contains (8450 bp) with 15 SSR incidence. The result indicates that there is no correlation between genome size and SSR incidence. RA and RD of SSR ranged from 1.14 to 4.34 and 7.89 to 29.87, while RA and RD of cSSR ranged from 0 to 0.5 and 0 to 8.23. Highly variation has been observed in RA and RD value of SSR and cSSR.

The most abundant repeat motif is “C” comprising of around 58% (126 of 219) of the mono-nucleotide SSRs. “G” is the second most prevalent repeat motif (70) incidence. Similarly, among the di-nucleotide repeats, the most prevalent motif was AC/CA comprising around 27% (240 of 884) followed by GT/TG (202 of 884), while TGA/ACT is the most prevalent motif of tri-nucleotide repeat incidence. Poly C/G is predominant in all the types of SSR (mono-tri) because of a high percentage of GC content in the studied genome. These results when looked at in terms of host range are significant as it has been reported that viruses tend to have predominantly mono-nucleotide SSRs in the A/T region [10, 17]. These results were not absolute suggesting the interplay of other factors in host determination. Also, in genomes with higher GC content, there have been some deviations reported [18]. The present dataset adds to that dimension that with varying GC content, the motif constitution of microsatellites would differ and may have other differential factors for host determination.

As discussed above in the microsatellite composition section, genomes with the same microsatellite incidence may also have a totally unique microsatellite signature in terms of composition and number of iterations. This is in concordance with previous reports on other virus families [10, 11, 19–21]. The role of SSRs in gene regulation, replication, protein function, biomarker, and genome evolution has been known making the variations in SSR in genomes of Caliciviridae an interesting platform.

We analyzed the distribution of SSR in the genome; ~ 90% of SSR incident were present in the coding regions, whereas only ~ 9% of SSR incident were found in the non-coding regions of the genome. SSR in the coding region are responsible for the genome diversity and evolution, while SSR in non-coding region are responsible for gene regulation and formation of a novel gene. In the genomes wherein there was no non-coding regions, rather than looking at absolute incidence of SSRs, we compared the SSR density therein with the coding regions. Interestingly, 18 genomes had a higher density of SSRs in the non-coding regions. This may be an indication of the non-coding regions equally contributing to the course of evolution and might be future genes in new viruses.

We analyzed the distribution of SSR in the coding region. Polyprotein contains 65% of SSR (837) followed by non-structural protein in the second position with 10% of SSR (150). As evident, even in the members of the family, the genomes do not follow any pattern in SSR incidence across proteins, reinforcing the idea about a unique microsatellite signature for each genome. The location of incident SSRs in Caliciviridae genomes reiterates two important aspects. Firstly, the virus genome is mainly comprised of coding region, thus most of the SSR incident are present in the coding region of the genome. Secondly, as reported earlier, SSR plays an important role in gene expression and genome evolution, thus the position of SSR in the protein region is imperative which is approved by the data.

As per the previous report, human and related species of viruses has A/T rich mono-nucleotide SSR repeats in their genome [10, 11, 17]. However, presently studied genomes do not follow the said pattern. This can be primarily attributed to the GC-rich nature of the genomes. Most species exhibit mono-nucleotide repeats exclusively to the G/C region of the genome including where humans are host. Only CLV32 with cat as host has mono-nucleotide repeats exclusively to the A/T region of the genome. This may be an indication of the potentiality of host divergence. Thus, the host range is dependent upon various factors as revealed by the present data, and the composition of the genome should be analyzed properly for the establishment of any rule.

## Conclusions

The Caliciviridae genomes do not conform to any pattern of SSR signature in terms of incidence, composition, and localization. This unique property of SSRs plays an important role in viral evolution by acting as sites for genome diversity. Their presence in the different proteins means the microsatellite alterations can impact their function and thus aid evolution. The clustering of similar host in the phylogenetic tree is the evidence of the uniqueness of SSR signature. Though SSR signature plays a key role for all viral genome evolution, the mechanism through which it happens needs to be explored.

### Supplementary Information

**Additional file 1.** Genomes features and extracted microsatellites of Caliciviridae in the study.

**Additional file 2.** SSRs and cSSRs extracted from Caliciviridae genomes.

**Additional file 3.** SSR incidence, tract size, composition and location in Caliciviridae genomes.

**Additional file 4.** SSR density range of genes of Caliciviridae genomes.

## Data Availability

All data are provided in the manuscript and supplementary files.
